# Tissue-Specific Diversity of Group 2 Innate Lymphoid Cells in the Skin

**DOI:** 10.3389/fimmu.2022.885642

**Published:** 2022-06-09

**Authors:** Tetsuro Kobayashi, Kazuyo Moro

**Affiliations:** ^1^Laboratory for Innate Immune Systems, RIKEN Center for Integrative Medical Sciences (IMS), Yokohama, Japan; ^2^Laboratory for Innate Immune Systems, Department of Microbiology and Immunology, Graduate School of Medicine, Osaka University, Osaka, Japan; ^3^Laboratory for Innate Immune Systems, Immunology Frontier Research Center (iFReC), Osaka University, Osaka, Japan; ^4^Laboratory for Innate Immune Systems, Graduate School of Frontier Biosciences, Osaka University, Osaka, Japan; ^5^Integrated Frontier Research for Medical Science Division, Institute for Open and Transdisciplinary Research Initiatives (OTRI), Osaka University, Osaka, Japan

**Keywords:** skin, ILC2, diversity, allergy, dermatology

## Abstract

Since the discovery of group 2 innate lymphoid cells (ILC2s), their developmental pathways, mechanisms of activation and regulation, and immunological roles in the steady state and in disease have been reported in various organs. ILC2s, which produce large amounts of IL-5 and IL-13 in response to tissue-derived factors and are essential in inducing and promoting allergic inflammation, have also been found to play multifaceted roles in maintaining tissue homeostasis. While T cells respond to foreign antigens, the activation of ILC2s is regulated by various tissue-derived factors, including cytokines, lipids, hormones, and neurotransmitters, and ILC2s show different phenotypes depending on the tissue in which they are present. In this review, we discuss tissue-specific characteristics of ILC2s in the skin. ILC2s, as defined in the lungs, intestinal tract, and adipose tissue, cannot be directly applied to cutaneous ILC biology, because skin ILC2s exhibit different aspects in the expression patterns of cell surface markers, the response to tissue-derived cytokines and the functions in both steady-state and inflammation. The skin contains ILCs with features of both ILC2s and ILC3s, and the plasticity between ILCs complicates their characters. Furthermore, the epidermis, dermis, and subcutaneous tissues contain ILCs with different characteristics; their localization has expanded our understanding of ILC function. Single-cell RNA-seq technology has further elucidated the role of ILCs in human skin and disease pathogenesis. Overall, this review discusses the phenotypical and functional heterogeneity of skin ILCs reported in recent years and highlights future directions within the field of ILC biology.

## Introduction

The skin is the largest barrier organ in the body and harbors a variety of innate and adaptive immune cells with diverse functions. Langerhans cells, γδT cells, and memory T cells are tissue resident immune cells and have been reported to communicate with keratinocytes and other stromal cells, playing crucial roles in both inflammation and tissue maintenance in the skin. Innate lymphoid cells (ILCs), a previously unappreciated family of innate immune cells, have provoked a paradigm shift in our understanding regarding the roles of tissue-resident innate immune cells in the homeostatic maintenance of tissue physiology and in the ﻿ induction, regulation, and resolution of inflammation (1).

Group 2 ILCs (ILC2s) were first reported as cells that produce type 2 cytokines, such as IL-5 and IL-13, in response to IL-33 and IL-25 in the adipose tissue and small intestine during helminth infection (2, 3). Subsequently, ILC2s were found to be present in various organs, including the lungs, tonsils, liver and muscle where they play an essential role in the development of allergic inflammation, tissue repair, and regulation of metabolism (4). ILC2s are also found in the skin (5–7). Skin, lungs and intestines are barrier organs, continuously exposed to different external stresses (mechanical and chemical) and invading microorganisms; thus, they have developed specific response mechanisms against various stimuli. The highly sophisticated tissue specificity of ILC2s is not surprising.

Important trademarks for defining ILC2s include the expression of the master transcription factor GATA3 and the ability to produce the type 2 cytokines, such as IL-5 and IL-13. However, the detection of ILC2-specific cell surface markers is used in a more versatile method in flow cytometry analysis. Lung ILC2s have been expansively researched over the past decade. They can be clearly separated from other ILCs by the expression of IL-33R (ST2). The lineage^-^ Thy1.2^+^ ST2^+^ population has been commonly used for the stable detection of typical ILC2s in lungs by flow cytometry. In contrast, the definition of skin ILC2s varies from study to study, which has resulted in confusion. Early studies on skin ILC2s used ST2 as a cell surface marker for their detection (5). However, the number of ILC2s detected using ST2 as a marker was very low in the lineage^-^ Thy1.2^+^ population, and ILC2 research in the skin has been greatly hampered by the incomplete characterization of ILC2s. A recent comprehensive analysis of skin ILC subsets revealed that only a small fraction of ILC2s in the skin expresses ST2, and the true nature of skin ILC2s is being gradually revealed (8, 9).

## True Characterization of Skin ILC2

Advances in unsupervised transcriptome analysis have facilitated the detailed dissection of unknown immune cells. In particular, single-cell RNA-seq analysis has proven to be a powerful tool to comprehensively understand the characteristics and heterogeneity of immune cells in which cell surface markers have not been well defined. Single cell RNA-seq analysis of ILCs in different tissues, including the skin, lungs, and gut of mice expressing reporter alleles for IL-5 (*Red5*), revealed clear segregation of ILC2s by tissue, suggesting unique, tissue-specific characteristics of ILC2s (8). While *Gata3*, *Il7r*, and *Crlf2* (which encodes the TSLP receptor subunit TSLPR) are broadly expressed in ILC2s across tissues, expression of *Icos*, *Ccr6*, and *Itgae* is highly enriched in skin ILC2s. In contrast to high expression of *Il1rl1*, which encodes IL-33R, in lung and adipose tissue ILC2s, skin ILC2s show only minimal expression of *Il1rl1*. In addition, the expression of *Il17rb*, which encodes IL-25R, is abundant in gut ILC2s. These data demonstrate the tissue-organizing transcriptome identities of ILC2 subsets and differential expression of cell surface markers of ILC2s in each organ and also suggest the variable cytokine dependency of ILC2 activation.

Consistent with the analysis of IL-5-producing ILC2s, analysis of IL-13 reporter mice (*Il13*-DsRed) revealed that IL-13-producing skin ILC2s expressed ICOS and CD44, whereas expression of ST2, CD25, and KLRG1 was low (10). Half of IL-13-producing cells among skin immune cells are ICOS^+^ ILC2s, and the steady state production of IL-13 is necessary for the development of CD11b^low^ type 2 dendritic cells and for the induction of allergic Th2 responses. The results of two studies showed that IL-5 and IL-13-producing skin ILC2s express ICOS, but not ST2. It should be noted, however, that ICOS is also expressed in other subsets of skin ILCs (9, 10).

Cytokine expression of ILC2s has been analyzed using various methods including *ex-vivo* stimulation (PMA+Ionomycin Ior specific cytokines), reporter mice and RNAseq of sorted cells; each method has advantages and disadvantages. Whereas *ex-vivo* stimulation forces cells to produce cytokines, steady state production of cytokines can be evaluated in reporter mice. L-13 expression was detected in both *ex-vivo* stimulation (8, 9) and reporter mice (*Il13*-DR and *Smart13*) (8, 10), suggesting production of IL-13 from ILC2s in steady state and activation state.

The fact that ILC2s express various cytokine receptors in different tissues is important when considering the development of therapies based on monoclonal antibodies for the treatment of allergies. While the use of anti-IL-4/IL-13 receptor antibodies has shown dramatic response in the treatment of atopic dermatitis (11), the failure of an anti-IL-33 treatment has suggested that IL-33-ILC2 axis may not be central in the pathogenesis of the disease. However, given that the majority of ILC2s in the skin do not express the IL-33 receptor, this result could have been expected. The efficacy of an anti-IL-33 therapy for the treatment of asthma was adequate but slightly weaker than that of anti-IL-4/IL-13 receptor antibodies, suggesting that consideration of the expression of cytokine receptors by ILC2s is essential for effective treatment (12).

ICOS expression has also been observed in migrating skin ILC2s (13). Tracking the migration of ILC2s in IL33tg-Kikume Green-Red mice revealed the migration of ILC2s from the skin to lymph nodes in the model of IL-33-induced dermatitis. This indicated that ICOS was expressed in both resident and migrating ILC2s, whereas CD103 expression was limited to skin-resident ILC2s. ICOS is a costimulatory molecule belonging to the CD28 superfamily and the importance of ICOS: ICOS-ligand interactions for the survival and cytokine production of ILC2s have been described in the lungs (14). However, the role of ICOS in skin ILC2s requires further investigation.

## Layer-Specific Compartmentalization of Skin ILC Subsets

The skin exerts its barrier function through a multilayered structure comprised of three distinct anatomical compartments: the epidermis, dermis, and subcutis (or subcutaneous tissue). Each of these layers have distinct physiological functions and harbor resident immune cells. The epidermis, the outermost layer of the skin and the first line of physical and immunological defense, contains a unique subset of T cells and dendritic cells; Vγ5^+^ γδT cells (called dendritic epidermal T cells (DETCs) based on their morphological appearance) and Langerhans cells, respectively. The dermis primarily consists of dense connective tissues and contains an abundance of immune cells including αβ and γδ T cells, macrophages, dendritic cells, and mast cells. The subcutis is rich in adipose tissue and contains immune cells that interact with adipocytes. The layer-specific distribution of immune cells indicates that the immunological microenvironment differs in each layer, and it is therefore not surprising that ILC2s, which highly depend on tissue-derived factors, adapt their cellular phenotypes to the location in which they reside.

Analysis of ILCs in each skin layer revealed compartment-specific characteristics of ILCs (9). The ILC2 subsets in dermis and subcutis display different profiles. ILC2s that express ST2, CD25, Sca-1, and KLRG1, which are expressed on typical ILC2s in lungs, are enriched in the deepest layer of the skin, the subcutis. Considering that ILC2s contribute to adipose tissue metabolism (15, 16), the enriched distribution of ILC2s in subcutaneous tissue, which display a high adipose content, can be expected. Although the dermis is generally a major target for analysis in dermatology and subcutaneous tissue is removed from skin when the back skin of mice is analyzed, it can be an important reservoir for skin ILC2s. ICOS^+^ CCR6^+^ ILCs, on the other hand, are located in both the epidermis and dermis and exhibit ILC2 characters. Epidermal ILCs also display ILC3-like signature gene expression, such as *Rorc*, *Lta*, and *Tcf7*, in both bulk and single-cell RNA-seq analysis. ILC3s are abundant in mucosal tissues, the gut, and oral mucosa, which are constantly exposed to both resident and infectious microbes. IL-22-producing ILC3s are essential for the innate immune responses to fungi and extracellular bacteria as they promote the production of antimicrobial peptides from epithelial cells (17–20). The skin is also inhabited by microbiota and this balance needs to be properly maintained. Epidermal ILCs produce TNF and lymphotoxins and regulate the steady-state balance of the skin surface microbiota by inhibiting antimicrobial lipid production from the sebaceous glands (9). Although epidermal ILCs are ILC3-like cells as mentioned above, they express ILC2-signature genes such as *Il13* and *Il2*. Furthermore, while both epidermal and dermal ILCs express ICOS and CCR6, analysis of IL-5 and IL-13 reporter mice has revealed preferential expression of ICOS and CCR6 in skin ILC2s (8, 10). Therefore, it is probably difficult to divide cell types by the expression of cell surface markers, and the anatomical location may endow ILCs with unique site-specific functions. Despite of accumulating knowledge of ILC2s in murine skin, little is known about localization and cell surface markers of ILC2s in human skin. Immunohistochemical analysis of distribution of ILCs in human skin revealed ILC populations, mainly ILC1s and ILC3s in the upper dermis of healthy skin, and increased numbers of ILC2s in atopic skin (21). While increase of ILC2s in skin of patients with atopic dermatitis has been reported in other studies (7, 22, 23), their localization has not been extensively analyzed.

## Variety of ILC2 Activating Cytokines

The skin ILC2 landscape which has been unveiled by single cell transcriptome analysis not only facilitates flow cytometry detection of ILC2s by cell surface molecules, but also provides information regarding the cytokines that can activate ILC2s. In contrast to adaptive immune T lymphocytes, which require TCR-MHC class II interactions for their activation, ILC activation is directly triggered by tissue-derived factors that mount antigen-independent immune responses. Consistent with the marked expression of IL-33R in lung ILC2s, IL-33 is a key ILC2-activating cytokine in allergen-induced asthmatic airway inflammation (24, 25). As ST2 expression is limited to a subset of ILC2s located in subcutaneous adipose tissue (9), other cytokines may be involved in epidermal and dermal ILC2 activation.

The mechanism of ILC2 activation has been investigated in an atopic dermatitis model in which calcipotriol (MC903), a vitamin D3 analog, was applied to mouse skin. The results indicated that T lymphocytes were not necessary in MC903-induced dermatitis, but inflammation was markedly improved when ILC2s were removed by the administration of antibodies against CD25 or CD90.2 (5). Furthermore, deletion of RORα, which is necessary for the ILC2 development, significantly improved MC903-induced dermatitis (7), indicating that ILC2s are essential for MC903-induced atopic inflammation.

In a study using the same model, Kim et al. found that TSLP receptor-deficient (*Tslpr*^−/−^) mice showed a markedly reduced response of ILC2s and improvement in MC903-induced skin inflammation, whereas disruption of IL-33 (*Il33*^−/−^) or IL-25R (*Il17rb*^−/−^) did not affect the ILC2 responses (5). In contrast, Salimi et al. reported that deficiency of IL-25R (*Il17rb*^−/−^) or IL-33R (*Il1rl1*^−/−^) significantly reduced both dermatitis and ILC2 activation (7). Mice overexpressing the IL-33 gene (*Il33*) in keratinocytes (hK14mIL33tg) were also shown to develop atopic-like dermatitis with a significant increase in ILC2s. In this study, ILC2 depletion by *RORα*-deficient bone marrow transplantation markedly improved dermatitis, indicating that keratinocyte-derived IL-33 causes atopic inflammation through ILC2 activation (26). Furthermore, a mouse model of antigen-driven allergic skin inflammation showed different aspects of ILC2 response. Dermatitis induced by epicutaneous sensitization with ovalbumin was significantly reduced by keratinocyte-specific IL-25 depletion (*K14*-Cre *Il25*^flox/flox^). A study using IL-13 reporter mice showed that ILC2s serve as a major source of IL-13 and that *Rora*-Cre *Il17rb*^flox/flox^ mice, which selectively lack IL-25R expression in ILC2s, demonstrate significantly decreased OVA-induced inflammation, suggesting that IL-25 acts directly on skin ILC2s to promote IL-13 production (27).

Unbiased transcriptomic analysis also revealed that skin ILC2s preferentially express the gene encoding the IL-18 receptor 1 (*Il18r1*) and that supplementation with TSLP and IL-18 induces a large amount of IL-13 production from skin ILC2s *in vitro*. In addition, IL-13 production in MC903-induced dermatitis was significantly reduced in IL-18-deficient mice (8). Consistent with this report, mice overexpressing murine *Il18* in keratinocytes under the control of the human keratin 14 promoter (KIL-18Tg) developed atopic dermatitis-like inflammation and type 2 inflammation independent of IgE/IgG1, suggesting that IL-18 induces type 2 inflammation through activation of ILC2s (28). On the other hand, in the lungs, Il18r1^+^ Tcf7^+^ ILCs exhibit capabilities of progenitors that can differentiate into ST2^+^ ILC2s and ST2^-^ non-ILC2s (29). It is not yet understood whether IL-18R^+^ skin ILC2s also have progenitor-like properties, and how tissue-specific effects of IL-18 in skin and lung ILC2s are established.

Skin ILC2s exhibit different cytokine reactivity depending on the dermatitis models. Further, mouse genetic background may influence the results; TSLP for ILC2 activation in MC903-inflammatin was evaluated in the C57BL/6 strain (5), whereas the IL-25 and IL-33 were analyzed in the BALB/c strain (7). It is also unclear whether ILC2s that are responsive to IL-33, IL-25, TSLP, and IL-18 belong to different subsets. Therefore, it may not be feasible to identify a single cytokine that activates skin ILC2s. ILC2s can be flexibly activated in response to changes in the surrounding environment and various external factors that invade the skin. It would thus be interesting to determine the conditions under which TSLP, IL-33, IL-25, and IL-18 are produced by epithelial and other stromal cells, leading to the activation of ILC2s, and whether ILC2 stimulated by different cytokines contribute to different aspects of innate immunity. Furthermore, it should be noted that the receptors for these cytokines are expressed in various immune cells including T cells, basophils, and macrophages. For example, TSLP elicits IL-4 production of basophils, which subsequently promotes ILC2 responses (22). Therefore, it may not be sufficient to understand only direct action of cytokines on ILC2s; instead, it is important to reveal a network of cytokines in different subsets of immune cells.

Nevertheless, because these cytokines serve as therapeutic targets for the treatment of atopic dermatitis and the monoclonal antibodies to these cytokines have been studied in clinical trials (30), it is important to investigate their differential roles and redundancies in ILC2 activation. In translational research, we need better understanding of the receptor expression profiles on ILC2s in human skin.

## Mixed ILC2–ILC3 Profiles in the Skin

Effector conversion among ILC subtypes during inflammation also contributes to the diversity of ILC2 composition, allowing flexible immune functions in the skin. ILCs are highly plastic and can differentiate from one subset into another. For example, human fetal intestine ILC3s can differentiate into ILC1s under the influence of IL-12 (31). IL-12 also induces the conversion of IL-1β activated human peripheral blood ILC2s to ILC1s (32, 33). Human tonsil ILC1s can also differentiate into ILC3s in the presence of IL-2, IL-23, and IL-1β (34). Immunological flexibility among ILC subsets may enhance tissue resilience but may simultaneously complicate disease pathology.

To characterize the potential transitions of skin ILC subsets, a study has been performed, which combines longitudinal scRNA-seq, scATAC-seq, *in vitro* experiments, and *in vivo* fate mapping in mouse models of psoriasis by injection of IL-23 or treatment with imiquimod (35). Since psoriasis is an IL-17-mediated inflammatory skin disease, increased ILC3s have been found in blood and skin of the patients (36, 37). In this study, IL-23 was found to trigger the conversion of tissue-resident skin ILCs, including quiescent-like cells and ILC2s, toward ILC3-like cells which are characterized by the co-production of IL-13 and IL-22 or IL-13 and IL-17A. This suggests effector transitions of ILCs and adaptation of mixed ILC2-ILC3 states. IL-23 is known to play a pivotal role in the expansion and survival of Th17, which has been considered a central effector in the pathogenesis of psoriasis (38). The conversion of ILC2s to IL-17-producing ILC3-like cells by IL-23 could be another important factor in this disease. Moreover, these findings underlined the limitations of the current standard experimental approaches that simply divide ILCs into three groups based on transcription factors and cytokine production and treat ILCs as discrete types. Tissues may have more partially committed cells that contribute to the flexibility of ILC responses.

Parasitic helminth infection or systemic IL-25 administration can induce a subset of migratory ILC2s that preferentially express the IL-25 receptor but not ST2. These cells that co-express GATA3 and RORγt, and have the capacity to produce IL-17A, have been referred to as inflammatory ILC2s (iILC2s) (39). In the skin, a subset of ILCs, which express both GATA3 and RORγt and produce IL-5, IL-13, and IL-17, has also been described in the inflammatory condition. Analysis of mice that exhibited hair loss revealed that microbial dysbiosis triggered ILC2-mediated inflammatory destruction of hair follicles. iILC2s circulated in an S1PR1-dependent manner and infiltrated the skin *via* the CCL20-CCR6 axis (40). Although the impact of iILC2s has been demonstrated in anti-helminth immunity (41), the contribution of iILC2s, particularly the potential plasticity in skin inflammation, is yet to be investigated. Co-production of type 2 and 3 cytokines in iILC2s would be of great interest to better understand mixed type of immune responses in inflammatory diseases.

A subset of skin ILCs that express CCR6 and RORγt and expand under inflammatory conditions have also been reported upon chronic ultraviolet exposure. These UV induced-ILC3s produce IL-22 and are associated with UV-induced keratinocyte carcinogenesis (42). Although ICOS is highly expressed in skin-resident ILC2s, UV-induced RORγt^+^ ILC3s also expressed ICOS. It is unclear whether ICOS^+^ ILC2s can be converted into ICOS^+^ ILC3s and whether they share the characteristics of iILC2s. It would thus be interesting to study the fate of ILC2s under different inflammatory conditions and the factors that drive conversion of ILC phenotypes.

Mixed ILC2–ILC3 profiles are more evident among human skin ILCs. Isolated human dermal CD127^+^ CRTH2^+^ ILC2s produce IL-17 after exposure to the hyphae of *Candida albicans* present in cell suspensions cultured from dermal samples (43). In the human peripheral blood, c-Kit^+^ CCR6^+^ ILC2s express RORγt at the baseline and produce IL-17 after exposure to IL-1β and IL-23, which is associated with differentiation of ILC3s. Furthermore, IL-17-producing ILC3s found in lesions of psoriasis, a type 3 immune-mediated inflammatory skin disease, have been found to switch back to ILC2s. Phenotypic switching of ILC2s to ILC3-like cells and of ILC3s to ILC2s may explain the highly plastic nature of human ILC2s.

Single-cell RNAseq analysis of human skin in atopic dermatitis further revealed the heterogeneous composition of human skin ILC2s (23). The results indicated that a discrete ILC2 cluster expressing *GATA3* and *RORA* was segregated from the NK, ILC1, and ILC3 clusters. ILC2s co-expressing ILC3 markers *RORC* and *AHR* were found in healthy human skin and these co-expressing cells were increased in atopic skin. Consistent with the expression of transcription factors, atopic ILC2s expressed type 2 cytokines (i.e. *IL5* and *IL13*) as well as type 3 cytokines (i.e. *IL22* and *IL26*). Th2/Th17/Th22 cytokine elevation has been observed in a subset of patients with atopic dermatitis, and the IL-23-IL-17 axis and IL-22 have been targeted by monoclonal antibodies for the treatment of atopic dermatitis (44, 45). The presence of ILC2s co-producing type 2 and 3 cytokines in atopic skin might therefore explain mixed cytokine profiles in patients and indicates that targeting type 2 cytokines alone might not be sufficient for the effective treatment. The mechanisms of coexisting ILC2 and ILC3 features in a single cell type, and the upstream signals that trigger and sustain the mixed status, need to be determined.

## Conclusion

**Figure 1:**
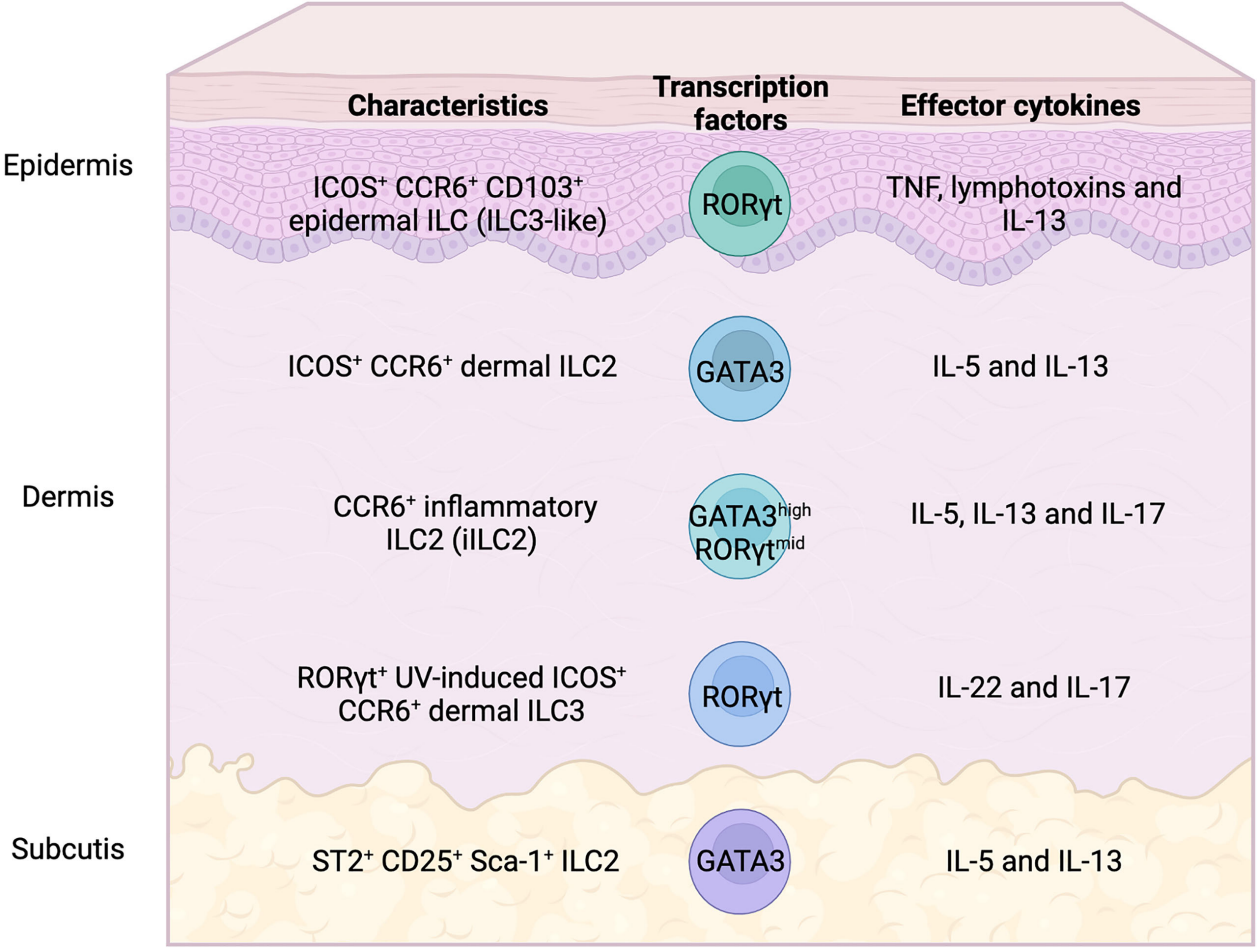
Heterogeneity of skin ILC subsets in mice. Skin ILC2s, which produce IL-5 and IL-13, express ICOS and CCR6, but not ST2 (IL-33 receptor), and play an important role in atopic and allergic inflammation. ST2^+^ ILC2s are only found in subcutaneous adipose tissues. Epidermal ILCs also express ICOS and CCR6, display the characteristics of both ILC2 and ILC3, and regulate microbiome balance. Inflammatory ILC2s that express both GATA3 and RORγt produce IL-5, IL-13, and IL-17, which infiltrate the tissues under inflammatory conditions. IL-22- and IL-17-producing RORγt+ ILC3s, which expand in UV-induced inflammation, also express ICOS and CCR6.

Over the past decade, our knowledge of ILC2s in the lungs and intestinal tract has been considerably expanded. In contrast, our understanding of the biology of ILC2s in the skin remains limited. ILCs have been commonly shown to demonstrate highly tissue-specific cellular characteristics that allow them to adapt to their surrounding environment. ILC2s play an important role in enhancing immune fitness of tissues locally by producing appropriate cytokines and modulating other immune and non-immune cells. This mini review discusses the highly heterogeneous features of skin ILC2s (summarized in **Figure 1** and **Table 1**). The heterogeneity and plasticity of skin ILC2s enable tissue-specific adaptation and might be related to the complex pathogenesis of inflammatory skin diseases, such as atopic dermatitis and psoriasis. Therefore, future research should not only use the experimental approaches that define ILCs as discrete subsets by cell surface markers, but rather require global and comprehensive analyses considering that ILCs are more diverse than previously estimated. Flow cytometry and microscopic analysis have accelerated the classification of immune cells over the past years. However, the limited numbers of parameters and our prior knowledge of cell-type definition have always posed as limiting factors in the study of the immune system complexity. Technological advancements of single cell analysis in transcriptome, epigenome, and proteome enable identification of cellular heterogeneity in far greater detail than conventional methods. Although the single cell analysis still has opportunities for improvement such as costs, technical accessibility, and depth of analysis, the unbiased character of single cell analysis may break through the bottleneck of ILC research.

**Table 1:** Heterogeneity of skin ILC2.

Cell identity			
Cell types	Detection	Characteristics	References
**Mouse**			
ILC2	Flow cytometry	Detected by expression of ST2 and CD25 Detected by expression of IL-7R, ICOS and c-Kit	(5, 46) (7)
IL-13 producing ILC2	Flow cytometry in IL-13 reporter mice		(6)
IL-5 producing ILC2	Single cell and bulk RNAseq in IL-5 reporter mice	High expression of *Gata3*, *Il7r*, *Crlf2*, *Icos*, *Ccr6*, *Itgae*, *Il18r1*, low expression of *Il1rl1*	(8)
IL-13 producing ILC2	Flow cytometry in IL-13 reporter mice	High expression of ICOS, low expression of ST2	(10)
Epidermal ILC Dermal ILC	Single cell and bulk RNAseq and flow cytometry	High expression of CCR6, CD103, *Rorc*, *Lta*, *Il13* (ILC3-like) High expression of ICOS, *Gata3*	(9)
ST2^+^ ILC2 in subcutis		High expression of ST2, CD25, Sca-1, KLRG1, *Gata3*, *Il5*	
Inflammatory ILC2	Single cell RNAseq and flow cytometry	Expression of GATA3, RORγt, CCR6, production of IL-5, IL-13 and IL-17A	(40)
**Human**			
ILC2	Flow cytometry	Detected by expression of ST2 and CD25 Detected by expression of IL-7Ra and CRTH2	(5, 22) (7)
ILC2	Immunohistochemistry	Expression of GATA3, IL-7Ra and CRTH2	(21)
ILC2	Single cell RNAseq	Expression of *GATA3* and *RORA*	(23)
Mixed ILC2 and ILC3		Coexpression of *GATA3*, *RORC*, *AHR*, *IL5*, *IL13*, *IL22*, *IL26* (increase in atopic skin)	
**Cytokine response**			
**Activating cytokines**	**Mice**	**Models**	**References**
TSLP	*Tslp*^−/−^ (C57BL/6)	MC903 atopic-like dermatitis	(5)
IL-33	*Il1rl1*^−/−^ (BALB/c) *Il1rl1*^−/−^ hK14mIL33tg (C57BL/6)	MC903 atopic-like dermatitis Wound healing IL-33 overexpression in keratinocytes	(7) (46) (26)
IL-25	*Il17rb*^−/−^ (BALB/c)	MC903 atopic-like dermatitis	(7)
	*K14*-Cre *Il25*^flox/flox^ (C57BL/6) *Rora*-Cre *Il17rb*^flox/flox^ (BALB/c)	OVA allergic skin inflammation	(27)
IL-18	*Il18*^−/−^ (C57BL/6) KIL-18Tg (C57BL/6)	MC903 atopic-like dermatitis IL-18 overexpression in keratinocytes	(8) (28)

## Author Contributions

TK and KM wrote the manuscript. All authors contributed to the article and approved the submitted version.

## Funding

This study was supported by KAKENHI (grant numbers 20H03705 and 20K21534 to TK, 18H05286 to KM) from the Japan Society for the Promotion of Science, AMED (grant number JP21gm6510005 to TK) and LEO Foundation Grant to TK.

## Conflict of Interest

The authors declare that the research was conducted in the absence of any commercial or financial relationships that could be construed as a potential conflict of interest.

## Publisher’s Note

All claims expressed in this article are solely those of the authors and do not necessarily represent those of their affiliated organizations, or those of the publisher, the editors and the reviewers. Any product that may be evaluated in this article, or claim that may be made by its manufacturer, is not guaranteed or endorsed by the publisher.
